# The Role of Urocortin 2 in Arterial Stiffness and Hypertension in Children with Obesity

**DOI:** 10.3390/children13101427

**Published:** 2026-10-18

**Authors:** Ulviye Kırlı, Nurcan Cengiz, Haşim Olgun, Sibel Tiryaki, Ercan Saruhan, Özlem Sangün, Kürşad Tosun

**Affiliations:** 1 Department of Pediatric Cardiology, Muğla Research and Training Hospital, 48000 Muğla, Türkiye; 2 Department of Pediatric Nephrology, Faculty of Medicine, Muğla Sıtkı Koçman University, 48000 Muğla, Türkiye; nurcandinler@mu.edu.tr; 3 Department of Pediatric Cardiology, Faculty of Medicine, Muğla Sıtkı Koçman University, 48000 Muğla, Türkiye; 4 Department of Medical Biochemistry, Faculty of Medicine, Muğla Sıtkı Koçman University, 48000 Muğla, Türkiye; ercansaruhan@mu.edu.tr; 5 Department of Pediatric Endocrinology, Faculty of Medicine, Başkent University, 01000 Adana, Türkiye; 6 Department of Mathematics and Statistics, Siena University, Loudonville, NY 12211, USA

**Keywords:** childhood obesity, urocortin 2, arterial stiffness, pulse wave velocity, hypertension, cardiovascular risk, vascular remodeling

## Abstract

**Highlights:**

**What are the main findings?**

- Children with obesity had higher pulse wave velocity and central systolic blood pressure, with a greater prevalence of hypertension than healthy controls.
- Serum urocortin 2 was independently and inversely associated with pulse wave velocity, but not with hypertension status.

**What are the implications of the main findings?**

- Urocortin 2 may be relevant to the relationship between childhood obesity and arterial stiffness, independently of obesity and hypertension status.
- Longitudinal and mechanistic studies are needed to clarify the clinical significance and potential biological role of urocortin 2 in pediatric vascular dysfunction.

**Abstract:**

**Background**: Childhood obesity is associated with hypertension (HT), arterial stiffness, and increased cardiovascular risk. Urocortin 2 (UCN2), a member of the corticotropin-releasing factor family, exerts vasodilatory and cardioprotective effects; however, its role in obesity-related vascular dysfunction in children remains unclear. We aimed to investigate the relationship between circulating UCN2 levels, HT, and arterial stiffness parameters in children with obesity. **Methods**: This prospective, single-center, cross-sectional case-control study included 154 children aged 10–18 years: 95 with obesity and 59 healthy controls. Anthropometric measurements, serum UCN2 concentrations, and metabolic parameters were assessed. Brachial-based oscillometric pulse wave analysis was performed to obtain estimated pulse wave velocity (PWV), augmentation index corrected for a heart rate of 75 beats/min (AIx@75), central systolic blood pressure (cSBP), and other hemodynamic parameters. Multivariable linear regression analyses were used to identify independent predictors of UCN2 and arterial stiffness. **Results**: Children with obesity had significantly higher PWV, cSBP, pulse pressure, cardiac output, and HT prevalence compared with controls (all *p* < 0.001). Although UCN2 levels tended to be lower in children with obesity, the difference was not statistically significant (*p* = 0.196). In children with obesity, UCN2 was negatively correlated with body mass index (*r* = −0.31, *p* = 0.003) and pulse pressure (*r* = −0.22, *p* = 0.031), and log-transformed UCN2 levels were negatively correlated with PWV (*r* = −0.22, *p* = 0.034). The independent negative association between UCN2 concentrations and PWV indicates an inverse relationship with arterial stiffness in childhood obesity. However, further longitudinal and mechanistic studies are required to determine whether this association reflects a underlying physiological involvement or therapeutic target. **Conclusions**: Childhood obesity is associated with adverse vascular alterations and increased arterial stiffness. The independent inverse association observed between UCN2 and PWV highlights a biological link with vascular stiffness in childhood obesity. However, whether UCN2 possesses biomarker utility or therapeutic relevance remains speculative and warrants evaluation in prospective longitudinal and clinical studies.

## 1. Introduction

Obesity and obesity-related comorbidities have become a major and alarming public health concern worldwide, affecting not only adults but also children and adolescents [1]. In 2019, the number of children and adolescents aged 5–19 years living with obesity was estimated at 158 million globally and is projected to increase to approximately 254 million by 2030 [2]. Since obesity in childhood frequently persists into adulthood, early intervention and effective management are of paramount importance to prevent long-term health consequences [3]. Childhood obesity is strongly associated with an increased risk of cardiovascular morbidity and mortality [1]. Among obesity-related cardiovascular complications, hypertension (HT) is often the earliest clinical manifestation and is increasingly diagnosed at younger ages [4]. Previous studies have demonstrated that weight gain is positively associated with blood pressure levels and that the prevalence of HT is substantially higher in children with obesity than in their non-obese peers [1,5,6]. The pathophysiology of obesity-related HT is multifactorial and involves sodium retention, overactivation of the renin-angiotensin-aldosterone system, increased sympathetic nervous system activity, altered vascular function, insulin resistance, and chronic low-grade inflammation [1,7]. Obesity and HT are also major risk factors for endothelial dysfunction and atherosclerosis. Obesity-induced chronic low-grade inflammation and oxidative stress contribute to vascular endothelial dysfunction, leading to early vascular damage and accelerated vascular aging [8]. Arterial stiffness is recognized as an early and reliable marker of vascular dysfunction and an independent predictor of future cardiovascular events [9]. Pulse wave velocity (PWV), aortic augmentation index (AIx), and central systolic blood pressure (cSBP), which can be obtained noninvasively using oscillometric techniques, are considered among the most appropriate and widely used parameters for the assessment of arterial stiffness [10,11]. Increasing evidence suggests that alterations in these parameters may already be present during childhood obesity, even before overt cardiovascular disease becomes clinically apparent. Complex biological mechanisms regulating energy homeostasis and food intake play a crucial role in the development and progression of obesity [12]. Central to these regulatory pathways are neuropeptides belonging to the corticotropin-releasing factor (CRF) family, including corticotropin-releasing hormone (CRH), urocortins (UCN1, UCN2, and UCN3), and their receptors, CRF receptor type 1 (CRHR1) and type 2 (CRHR2). These neuropeptides have been shown to participate in the regulation of energy balance and appetite suppression in mammals [13]. Beyond their metabolic effects, urocortins are widely expressed in the cardiovascular system, including cardiomyocytes, vascular smooth muscle cells, and endothelial cells, where they exert important physiological actions such as vasodilation, reduction in peripheral vascular resistance, positive inotropic and lusitropic effects, and protection against ischemia-reperfusion injury [14,15]. Among the urocortin family members, UCN2 has attracted increasing attention because of its potent cardiovascular effects mediated predominantly through CRHR2. Experimental studies have demonstrated that UCN2 exerts potent hemodynamic effects, inducing sustained inhibition of cardiac sympathetic nerve activity alongside blood pressure reduction [16]. Furthermore, CRHR2 stimulation by UCN2 has been associated with beneficial effects on blood pressure regulation, left ventricular dimensions, cardiac function, and vascular homeostasis, suggesting that UCN2 may represent a promising therapeutic target for arterial HT and cardiovascular diseases [17,18]. Despite these findings, the relationship between circulating UCN2 levels and early vascular alterations, particularly arterial stiffness, in children with obesity remains poorly understood. Therefore, in the present study, we aimed to investigate the association of serum UCN2 levels with HT and oscillometric arterial stiffness parameters, including PWV, AIx, and cSBP, in children with obesity. We hypothesized that alterations in circulating UCN2 levels may be associated with increased arterial stiffness and elevated blood pressure, thereby contributing to the early cardiovascular risk profile observed in childhood obesity.

## 2. Methods

### 2.1. Study Group

This was a prospective, single-center, cross-sectional case-control study conducted at the Pediatric Outpatient Clinic of Muğla Sıtkı Koçman University between January 2020 and June 2022. Children aged 10–18 years were enrolled in the study. Only children with newly diagnosed primary obesity were included in the group with obesity. Participants with a history of pharmacological treatment within the previous month, active or chronic infection, or any concomitant chronic disease were excluded. The control group consisted of healthy, normal-weight, age-matched children who were recruited voluntarily from the general pediatric outpatient clinic. The study protocol was approved by the Muğla Sıtkı Koçman University Clinical Research Ethics Committee (Decision No: 01/VII, Date: 1 March 2018). Written informed consent was obtained from all parents or legal guardians, and assent was obtained from the children whenever appropriate. All participants and their guardians were informed about the purpose and procedures of the study. The study was conducted in accordance with the ethical principles of the Declaration of Helsinki.

### 2.2. Anthropometric Measurements

All children underwent a standardized physical examination performed by the same pediatrician. Anthropometric measurements were obtained according to the recommendations of the World Health Organization (WHO). Body weight was measured using a calibrated physician scale, and height was measured using a wall-mounted stadiometer, with participants wearing light clothing and no shoes. All measurements were performed using the same equipment. Body mass index (BMI) was calculated as weight (kg) divided by height squared (m^2^) and was retained as a continuous variable in the correlation and regression analyses. BMI percentiles were determined according to the Centers for Disease Control and Prevention growth charts and categorized as follows: underweight (<5th percentile), normal weight (5th–84th percentile), overweight (85th–94th percentile), and obese (≥95th percentile) [19]. Age- and sex-specific BMI percentiles were therefore used for the classification of weight status, whereas absolute BMI was used as a continuous measure to quantify adiposity in the regression analyses. Pubertal status was not systematically recorded in the study dataset and was therefore not available for adjustment in the statistical analyses. Similarly, while participants were age-matched between groups, age and sex were evaluated as potential confounders in initial model exploration; however, because the study population was tightly age-matched (mean age 13.22 ± 2.47 vs. 13.22 ± 2.40 years, *p* = 0.995) and sex distribution was balanced (36% vs. 39% female, *p* = 0.885), their inclusion did not alter the primary associations and they were omitted from final parsimonious models to avoid over fitting.

### 2.3. Twenty-Four-Hour Ambulatory Blood Pressure Monitoring and Pulse Wave Analysis

Twenty-four-hour ambulatory blood pressure monitoring (ABPM) and pulse wave analysis were performed using the Mobil-O-Graph^®^ Pulse Wave Analysis Monitor (I.E.M. GmbH, Stolberg, Germany). This validated oscillometric device provides non-invasive assessment of peripheral and central blood pressure parameters, including central systolic blood pressure (cSBP), pulse wave velocity (PWV), augmentation pressure (AP), augmentation index (AIx), augmentation index corrected for a heart rate of 75 beats/min (AIx@75), cardiac output (CO), peripheral resistance (PR), and reflection coefficient (RC) [20]. The device estimates the central aortic pressure waveform from brachial oscillometric recordings using a transfer function algorithm. AIx@75 was calculated as the ratio of augmentation pressure to central pulse pressure and expressed as a percentage (AIx@75 = AP/cPP × 100). The Mobil-O-Graph device was fitted in the morning, and participants continued their routine daily activities throughout the 24 h monitoring period. Blood pressure measurements were obtained every 20 min during daytime and every 30 min during nighttime. Recordings with more than 20 valid measurements in each recording period and at least 18 h of valid recordings were considered acceptable for analysis. HT was defined according to the 2017 American Academy of Pediatrics Clinical Practice Guidelines [21]. In children younger than 13 years, stage 1 HT was defined as mean systolic blood pressure (SBP) and/or diastolic blood pressure (DBP) between the 95th percentile and the 95th percentile plus 12 mmHg for age, sex, and height, whereas stage 2 HT was defined as SBP and/or DBP ≥95th percentile plus 12 mmHg. In children aged 13 years or older, stage 1 HT was defined as SBP/DBP between 130/80 and 139/89 mmHg and stage 2 HT as SBP/DBP ≥140/90 mmHg. Based on 24 h ABPM, children were classified as hypertensive when more than 25% of valid systolic and/or diastolic blood pressure measurements exceeded the 95th percentile. It should be noted that office-based HT staging in our study was defined according to the 2017 American Academy of Pediatrics (AAP) guidelines based on percentiles and fixed thresholds, whereas ambulatory HT was defined using α >25% blood pressure load threshold. Although earlier pediatric guidelines incorporated blood pressure load into ABPM criteria, the updated 2022 American Heart Association (AHA) scientific statement on pediatric ABPM no longer recommends blood pressure load for ambulatory HT classification, favoring mean ambulatory blood pressure levels instead. To address potential diagnostic discrepancies between legacy thresholds and current recommendations, a post hoc reconciliation of our ABPM dataset was conducted. Re-evaluating our cohort using the updated 2022 AHA mean ambulatory blood pressure thresholds (≥95th percentile or ≥130/80 mmHg), 41 of the 45 participants (91.1%) initially classified as hypertensive under the >25% blood pressure load criterion maintained their hypertensive status. Four participants with borderline load values fell below the mean-based threshold, confirming high concordance while demonstrating that mean-based classification provides a slightly more conservative diagnostic yield.

### 2.4. Sample Collection and Blood Analysis

Following an overnight fast of at least 8 h, venous blood samples were collected from all participants for the measurement of fasting plasma glucose, insulin, and serum UCN2 concentrations. Insulin resistance was assessed using the Homeostasis Model Assessment of Insulin Resistance (HOMA-IR), calculated as: HOMA-IR = fasting insulin (μU/mL) × fasting glucose (mg/dL)/405 [22]. For serum UCN2 analysis, blood samples were allowed to clot at room temperature and centrifuged approximately 30 min after collection. Serum aliquots were separated and stored at −80 °C until analysis. Serum UCN2 concentrations were measured according to the manufacturer’s instructions at the Biochemistry Laboratory of Muğla Sıtkı Koçman University Faculty of Medicine using a commercially available Human Urocortin 2 ELISA kit (Phoenix Pharmaceuticals, Inc., Burlingame, CA, USA; Catalog No. EK-019-30).

### 2.5. Variables and Definitions

Continuous hemodynamic variables included PWV, AIx@75, peripheral systolic blood pressure (pSBP), peripheral diastolic blood pressure (pDBP), cSBP, central diastolic blood pressure (cDBP), pulse pressure (PP), mean arterial pressure (MAP), CO, PR, and RC. Anthropometric status was represented by BMI, whereas metabolic status was assessed using HOMA-IR. Serum UCN2 concentration (ng/mL) was the primary biomarker of interest.

### 2.6. Statistical Analysis

All statistical analyses were performed using *R* software (version 4.3.1). Continuous variables are presented as mean ± standard deviation (SD) or median (interquartile range), depending on data distribution, whereas categorical variables are expressed as frequencies and percentages. Between-group comparisons were performed using *Welch’s t*-test for normally distributed variables or the *Wilcoxon-Mann-Whitney* test for non-normally distributed variables. Categorical variables were compared using the *Chi-square* test with *Yates’* continuity correction. *Pearson* correlation coefficients were calculated separately for obese and non-obese children to evaluate linear associations among study variables. Model specifications and variable coding were defined as follows: continuous variables were included in their original metrics, with the exception of serum UCN2 when evaluated for correlation with PWV, where log-transformation was applied to satisfy linearity assumptions. In all multivariable regression models, categorical variables were dummy-coded (Obesity status: 0 = non-obese, 1 = obese; HT status: 0 = normotensive, 1 = hypertensive). Missing data handling was managed via complete-case analysis, as all 154 participants had complete records across the analyzed variables. Multicollinearity was evaluated using variable-specific Variance Inflation Factors (VIFs), with all final model predictors demonstrating VIF values well below 2.5 (specifically: UCN2 VIF = 1.12, Obesity status VIF = 2.10, HT status VIF = 1.45, cSBP VIF = 1.85, and BMI VIF = 2.20). Log transformation was applied to UCN2 values when assessing its association with PWV to improve linearity and satisfy model assumptions. Multivariable linear regression analyses were used to investigate the independent effects of obesity and HT on hemodynamic and vascular parameters, to identify independent predictors of UCN2 concentration, and to determine whether UCN2 was independently associated with PWV and AIx@75. In the models evaluating the independent effects of obesity and HT, obesity status and HT status were considered as predictor variables, while PWV, PP, CO, and UCN2 concentrations were evaluated as dependent variables. In the model evaluating independent predictors of UCN2 concentration, PWV, BMI, cSBP, PR, and CO were considered as candidate predictors. To evaluate the independent association of UCN2 with arterial stiffness, an additional multivariable linear regression model was fitted with PWV as the dependent variable, incorporating UCN2, obesity status, HT status, cSBP, and BMI as covariates. To minimize multicollinearity, variables that were intrinsically related were not included in the same model. Specifically, pSBP, pDBP, cDBP, PP, and MAP were excluded in favor of cSBP, whereas weight and height were excluded in favor of BMI. Obesity status was excluded from the UCN2 model because it represents a dichotomized form of BMI. Final models were selected using a stepwise procedure based on the Akaike Information Criterion (AIC). Variance inflation factors were calculated to assess multicollinearity among predictors in the final models. Given the strong bivariate correlation between PWV and cSBP, multicollinearity was rigorously tested using VIFs. All predictor variables in the final multivariable models demonstrated VIF values below 2.5, well below the conservative threshold of 5, confirming that multicollinearity did not distort the regression estimates. Stepwise predictor selection was conducted based on the AIC to identify the most parsimonious model that optimizes goodness-of-fit while minimizing parameter redundancy. For multiple univariate pairwise group comparisons across subgroups, *p*-values were adjusted using the *Benjamini-Hochberg* false discovery rate (FDR) procedure to control for Type I error accumulation. To formally test whether the association between UCN2 and PWV differed significantly between children with obesity and healthy controls, a multiplicative interaction term (UCN2 × Obesity status) was included in the multivariable linear regression model. Urocortin 2 was log-transformed only when its association with PWV was assessed by correlation analysis, because log transformation improved linearity and better satisfied the assumptions of the analysis. Urocortin 2 values were not log-transformed in the multivariable model presented in Table 1. Model fit was assessed using R-squared and adjusted R-squared values. A two-sided *p*-value < 0.05 was considered statistically significant. The multivariable regression model for PWV was performed in the entire study population, including all 154 participants, and included obesity status and HT status as covariates. Although both continuous metrics (BMI, cSBP) and their corresponding clinical threshold categories (obesity status, HT status) capture related physiological dimensions, they were retained simultaneously to evaluate both the linear gradient effect and the distinct clinical diagnostic threshold risk. To address potential concerns regarding redundant variance, model stability was verified using VIFs, which remained well below conservative thresholds (all VIFs < 2.5). Furthermore, a parsimonious sensitivity model incorporating exclusively continuous predictors alongside UCN2 yielded consistent findings (Table 2).

## 3. Results

**Figure 1:**
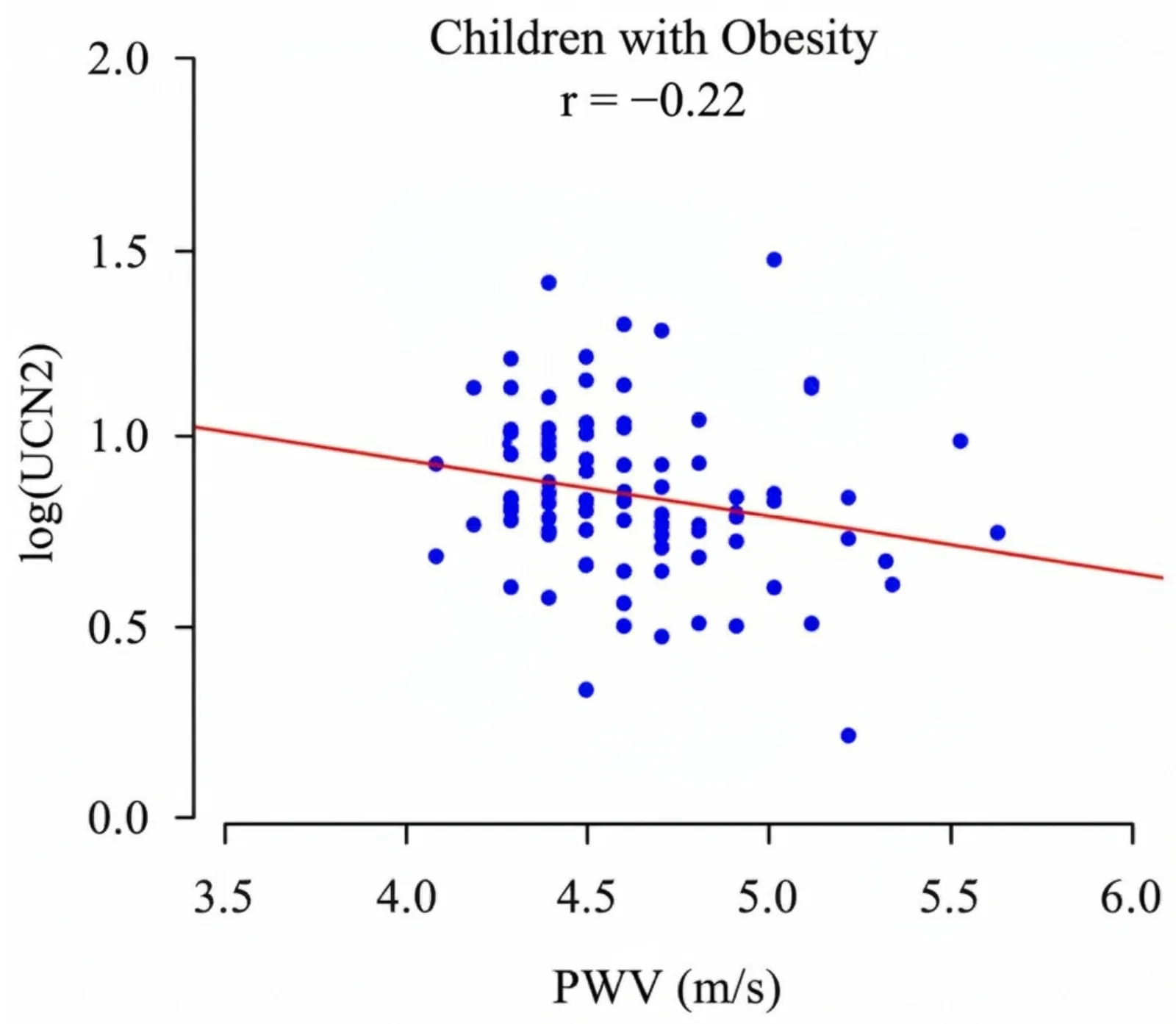
Scatter plot of PWV against log-transformed UCN2 in children with obesity, illustrating the negative correlation (*r* = −0.22, *p* = 0.034).

The study population consisted of 154 children aged 10–18 years, including 95 children with exogenous obesity and 59 healthy controls (see Supplementary Table S1 for details). Females comprised 36% of the group with obesity and 39% of the control group (*p* = 0.885). The mean age was 13.22 ± 2.47 years in the group with obesity and 13.22 ± 2.4 years in the control group (*p* = 0.995). Compared with controls, children with obesity had significantly higher PWV, pSBP, cSBP, PP, and CO (all *p* < 0.001). Although serum UCN2 levels were lower in children with obesity than in controls, the difference did not reach statistical significance (*p* = 0.196) (Table 3). Of the 154 participants, 45 (29%) were hypertensive. Hypertension was detected in 38 of 95 children with obesity (40%) and in 7 of 59 controls (11.8%), corresponding to a significantly higher prevalence in the group with obesity (*p* = 0.0004). Serum UCN2 levels tended to be lower in hypertensive children (2.22 ± 0.59 ng/mL) than in normotensive children (2.36 ± 0.45 ng/mL); however, this difference was not statistically significant (*p* = 0.156). Hemodynamic parameters and UCN2 levels were further compared between obese and non-obese children within hypertensive and normotensive subgroups (Table 4). Among hypertensive children, obese and non-obese participants differed significantly only with respect to HOMA-IR (*p* < 0.001), whereas no statistically significant differences were observed in hemodynamic parameters (all *p* > 0.05). In contrast, among normotensive children, participants with obesity exhibited significantly higher PWV (*p* < 0.001), cSBP (*p* = 0.008), PP (*p* < 0.001), CO (*p* < 0.001), and HOMA-IR (*p* < 0.001) than non-obese normotensive children. Serum UCN2 levels did not differ significantly between obese and non-obese children within either the hypertensive or normotensive groups (*p* = 0.397 and *p* = 0.521, respectively). Among the 45 hypertensive children, 29 had stage 1 HT (22 obese and 7 controls), whereas 16 children, all of whom were obese, had stage 2 HT. Mean PWV increased progressively with HT severity, from 4.41 ± 0.19 m/s in normotensive children to 4.73 ± 0.17 m/s in children with stage 1 HT and 5.04 ± 0.28 m/s in children with stage 2 HT. All pairwise comparisons were statistically significant (*p* < 0.001). No significant association was observed between HT stage and serum UCN2 levels (*p*-values > 0.05). Correlation analyses are presented in Table 5. In children with obesity, serum UCN2 levels showed weak but statistically significant negative correlations with BMI (*r* = −0.31, *p* = 0.003) and PP (*r* = −0.22, *p* = 0.031), whereas a weak positive correlation was observed with RC (*r* = 0.21, *p* = 0.041). Because the relationship between UCN2 and PWV was non-linear, UCN2 values were log-transformed before correlation analysis. Log-transformed UCN2 levels were negatively correlated with PWV in children with obesity (*r* = −0.22, *p* = 0.034) (Figure 1). In contrast, no significant correlation between UCN2 and PWV was observed in the control group (*r* = −0.14, *p* = 0.291). With respect to arterial stiffness measures, PWV was strongly and positively correlated with central and peripheral blood pressure, PP, MAP, and CO in both obese and non-obese children. The correlation between PWV and BMI was statistically significant in children with obesity (*r* = 0.42, *p* < 0.001) but not in controls (*r* = 0.24, *p* = 0.067). AIx@75 was positively correlated with peripheral vascular resistance and negatively correlated with CO in both groups. In children with obesity, AIx@75 also demonstrated a weak negative correlation with BMI (*r* = −0.23, *p* = 0.029) (Table 6). Multivariable linear regression analyses were performed to evaluate the independent associations of obesity and HT status with vascular and hemodynamic parameters (Table 7). For PWV, PP, and CO, both obesity and HT status emerged as statistically significant independent predictors. For PWV, obesity status (*β* = 0.124, *p* < 0.001) and HT status (*β* = 0.289, *p* < 0.001) were independently associated with the outcome in the initial dual-variable model (Table 7). In the fully adjusted multivariable model incorporating all covariates (Table 2), obesity status (*β* = 0.089, *p* = 0.011) and HT status (*β* = 0.245, *p* < 0.001) maintained their significant independent associations with PWV. In contrast, neither obesity (*β* = −0.065, *p* = 0.451) nor HT (*β* = −0.121, *p* = 0.192) was significantly associated with serum UCN2 levels. Independent predictors of serum UCN2 concentrations were further evaluated using multivariable linear regression (Table 1). Serum UCN2 levels were independently and negatively associated with PWV (*β* = −1.106, *p* = 0.004), BMI (*β* = −0.012, *p* = 0.022), and PR (*β* = −2.733, *p* = 0.010). Cardiac output demonstrated a borderline negative association with UCN2 levels (*β* = −0.531, *p* = 0.053), whereas cSBP was positively associated with UCN2 (*β* = 0.065, *p* < 0.001). HT status was not significantly associated with UCN2 levels in the multivariable model. Finally, an extended multivariable linear regression model was used to determine whether UCN2 was independently associated with arterial stiffness. In this model, serum UCN2 remained a significant negative predictor of PWV (*β* = −0.042, *p* = 0.008), independent of the other covariates included in the analysis. In this multivariable model evaluated among all study participants (*n* = 154), serum UCN2 concentrations remained a significant independent negative predictor of PWV (unstandardized coefficient *β* = −0.042, 95% confidence interval: −0.073 to −0.011, *p* = 0.008) after adjusting for obesity status (*β* = 0.089, 95% confidence interval: 0.021 to 0.157, *p* = 0.011), HT status (*β* = 0.245, 95% confidence interval: 0.178 to 0.312, *p* < 0.001), cSBP (*β* = 0.018, 95% confidence interval: 0.014 to 0.022, *p* < 0.001), and BMI (*β* = 0.005, 95% confidence interval: 0.001 to 0.009, *p* = 0.018). The detailed specifications, regression coefficients, 95% confidence intervals, and goodness-of-fit parameters for this principal model supporting the independent association between UCN2 and PWV are presented in Table 2. Furthermore, when testing for potential moderation, the interaction term between UCN2 and obesity status was not statistically significant (*β* = −0.038, *p* = 0.312), indicating that while bivariate correlations were statistically significant only in the obese cohort, the slope of the UCN2-PWV relationship did not significantly differ between the obese and control groups. In an age- and sex-adjusted sensitivity multivariable linear regression model for PWV, serum UCN2 remained a statistically significant independent negative predictor of PWV (*β* = −0.039, 95% CI: −0.069 to −0.009, *p* = 0.012) after further adjusting for age (*β* = 0.003, *p* = 0.420) and sex (*β* = −0.015, *p* = 0.510) alongside obesity status, HT status, cSBP, and BMI.

## 4. Discussion

Based on the available literature, this study appears to be among the first to explore the potential link between circulating UCN2 levels and arterial stiffness in a pediatric obese population. The principal findings of the present study are as follows: (i) children with obesity exhibited significantly higher systolic blood pressure, PP, CO, and PWV compared with healthy controls; (ii) the prevalence of HT was significantly higher in children with obesity; (iii) PWV was independently associated with both obesity and HT; (iv) serum UCN2 levels tended to be lower in children with obesity and BMI emerged as an independent negative predictor of UCN2; and (v) UCN2 was independently and negatively associated with PWV, suggesting a potential relationship between UCN2 and obesity-related vascular alterations. Childhood obesity is one of the most important risk factors for cardiovascular and metabolic diseases and has become a major public health challenge worldwide [1]. Numerous studies have demonstrated that obesity during childhood is associated with an increased risk of dyslipidemia, insulin resistance, non-alcoholic fatty liver disease, HT, and early cardiovascular disease [23,24]. In a recent study including 2917 preschool children, BMI and waist circumference were significantly associated with traditional cardiometabolic risk factors, indicating that the cardiovascular consequences of obesity may begin at an early age [24]. Similarly, Huang et al. reported that children and adolescents with overweight or obesity carry a substantial burden of cardiometabolic abnormalities, with approximately one-third exhibiting hepatic steatosis and more than 15% presenting with HT [23]. HT is considered the most common cardiovascular complication of childhood obesity and is increasingly diagnosed at younger ages [25]. Previous studies have reported HT prevalence’s ranging from 8.6% to 27.8% among children with obesity [5,6,26,27,28]. In a systematic review and meta-analysis including 47 studies, Song et al. demonstrated that HT was present in 15.3% of obese youths compared with only 1.9% of normal-weight children [6]. In the present study, the prevalence of HT in children with obesity was 40%, which is somewhat higher than that reported previously. Furthermore, our study utilized a >25% blood pressure load cutoff to define ambulatory HT based on traditional pediatric ABPM recommendations. As the updated 2022 AHA scientific statement has phased out blood pressure load in favor of mean ambulatory blood pressure percentiles, using the historical %25 load criterions may have contributed to a higher rate of ambulatory HT classification in our cohort compared with studies applying updated mean-based thresholds. The relatively high prevalence of HT observed in our cohort may partly reflect referral bias, as our institution is a tertiary referral center where children with more severe obesity and metabolic disturbances are more likely to undergo comprehensive cardiovascular evaluation. In addition, children with obesity exhibited significantly higher cSBP and PP than normal-weight children, whereas no significant difference was observed in diastolic blood pressure. These findings support the concept that obesity-related vascular alterations preferentially affect systolic and central hemodynamic parameters. Accumulating evidence indicates that obesity accelerates vascular aging and promotes early atherosclerotic changes even during childhood [29]. Because structural and functional vascular abnormalities precede clinically overt cardiovascular disease, arterial stiffness has emerged as an important surrogate marker of cardiovascular risk. Among the available noninvasive techniques, directly measured carotid-femoral pulse wave velocity (cfPWV) is widely considered the clinical gold standard for assessing central arterial stiffness [11]. In the present study, PWV was noninvasively estimated using a validated brachial cuff-based oscillometric device through a mathematical transfer function, which serves as a practical surrogate measure rather than direct carotid-femoral measurement. It is important to emphasize that cfPWV, conventionally assessed using applanation tonometry, remains the non-invasive reference standard for aortic stiffness assessment. Estimated PWV derived from single-site brachial oscillometric algorithms provides a practical and useful surrogate measure in pediatric settings, but it fundamentally differs from direct, dual-site cfPWV measurements and cannot be considered interchangeable with them. Recent meta-analysis by Cote et al., including 15 studies, demonstrated that obesity in children and adolescents is associated with increased arterial stiffness [30]. Likewise, Mihuta et al. reported significantly higher PWV values in children with obesity compared with healthy controls and found a positive correlation between PWV and BMI, suggesting that adiposity contributes directly to vascular stiffening [31]. Our findings are in agreement with these studies. PWV was significantly higher in children with obesity than in controls and showed a positive correlation with BMI only in the group with obesity. Moreover, multivariable analyses demonstrated that obesity and HT were independently associated with PWV. Several biological mechanisms may explain the link between obesity and arterial stiffness. Experimental studies have shown that obesity induces vascular remodeling through alterations in extracellular matrix composition, elastin fragmentation, and increased collagen deposition [32]. In both humans and animal models, obesity has been associated with downregulation of lysyl oxidase (LOX), a key enzyme responsible for elastin cross-linking, resulting in impaired arterial elasticity and increased PWV [33]. Furthermore, obesity-related chronic low-grade inflammation and oxidative stress promote endothelial dysfunction and vascular remodeling by stimulating matrix metalloproteinases and impairing nitric oxide bioavailability [34]. Taken together, these findings suggest that excess adiposity itself contributes to arterial stiffening through structural and functional changes within the arterial wall. In the present study, PWV was positively correlated with both central and peripheral blood pressure parameters. In addition, mean PWV increased progressively according to HT stage. When obese and non-obese children were analyzed separately, hypertensive children exhibited higher PWV values than normotensive children regardless of obesity status. These observations are consistent with previous reports. Kulsum-Mecci et al. demonstrated that children with combined obesity and HT had significantly higher PWV values than healthy controls, indicating that HT amplifies obesity-related vascular damage [35]. Nevertheless, the independent association of obesity with PWV observed in our study suggests that obesity itself contributes substantially to arterial stiffening, beyond the effects mediated by elevated blood pressure. AIx@75 is another widely used surrogate marker of arterial stiffness and wave reflection. However, the impact of childhood obesity on AIx@75 remains controversial. Mihuta et al. reported higher AIx@75 values in children with obesity [31], whereas other pediatric studies have observed altered wave reflection patterns and increased arterial stiffness parameters in children and adolescents with overweight and obesity [36,37,38]. In a large cohort of 6816 children, Jakab et al. found that AIx@75 was significantly lower in overweight and children with obesity than in normal-weight peers, although they observed no significant difference in PWV after strictly matching for age, sex, and systolic blood pressure [37]. In our study, AIx@75 values tended to be lower in children with obesity, although the difference did not reach statistical significance. Furthermore, AIx@75 showed a weak but significant negative correlation with BMI. Taken together, these findings suggest that obesity-related changes in wave reflection may involve complex alterations in vascular tone, peripheral resistance, and autonomic regulation rather than simply reflecting increased arterial stiffness. Recently, increasing attention has been focused on the role of the CRF family and its receptors in the regulation of energy homeostasis and cardiovascular function. Experimental studies have shown that urocortins participate in appetite suppression, energy expenditure, stress response, and inflammatory regulation [13,39]. In particular, CRHR2 signaling has been shown to play a key role in regulating feeding microstructure and meal size [40], and exogenous UCN2 administration reduces food intake in animal models [41]. Therefore, the UCN2/CRHR2 system has emerged as a potential therapeutic target for obesity and metabolic disorders. However, data regarding circulating UCN2 levels in childhood obesity are extremely limited. Kavalakatt et al. reported that UCN2 levels were significantly elevated in overweight children, whereas no difference was observed between obese and normal-weight children [42]. Similarly, Priego et al., demonstrated that children with higher BMI exhibited lower UCN2 gene expression levels and that frequent consumption of high-fat foods was associated with reduced UCN2 expression [43]. In our study, serum UCN2 levels were lower in children with obesity than in controls, although this difference did not reach statistical significance. Importantly, BMI emerged as an independent negative predictor of UCN2. This finding suggests that increasing adiposity itself may influence the regulation of the UCN2/CRHR2 system, even before clinically apparent cardiovascular complications develop. Taken together, these observations support the hypothesis that UCN2 may function as a compensatory neuropeptide during the early stages of weight gain, aiming to suppress food intake and counteract obesity-associated metabolic and cardiovascular disturbances. However, this compensatory response may become insufficient or dysregulated with increasing adiposity, ultimately contributing to the progression of obesity and its complications. UCN2 has also attracted considerable interest because of its cardiovascular actions. Experimental studies have demonstrated that UCN2 exerts potent vasodilatory effects, reduces peripheral vascular resistance, and lowers blood pressure through CRHR2-mediated pathways [17,32,44]. Liu et al. further showed that UCN2 improves systolic and diastolic function by inhibiting L-type calcium channels and promotes reverse ventricular remodeling, suggesting that it may represent a novel therapeutic target for HT [18]. To our knowledge, no previous study has investigated circulating UCN2 levels in hypertensive children. Available data are limited to adult populations and remain inconsistent. Aslan et al., found significantly higher UCN2 levels in hypertensive adults and identified UCN2 as an independent determinant of HT [45]. However, most participants in that study were receiving antihypertensive treatment, which may have influenced circulating UCN2 concentrations. In contrast, Dzieza et al., investigated newly diagnosed untreated hypertensive adults and demonstrated lower logUCN2 levels in hypertensive patients than in normotensive individuals, with higher UCN2 levels being associated with lower blood pressure values [46]. In the present study, although UCN2 levels tended to be lower in hypertensive children, neither HT status nor HT severity was independently associated with UCN2. Taken together, our findings do not support a direct relationship between HT and circulating UCN2 levels during childhood. In our cohort, circulating UCN2 concentrations showed a statistically significant inverse association with PWV, whereas no significant association was observed with HT status. While these cross-sectional findings highlight an association with arterial stiffness, they do not establish that UCN2 is selectively or more closely involved in vascular remodeling compared to blood pressure regulation. To our knowledge, no previous study has examined the relationship between circulating UCN2 levels and PWV in either children or adults. In subgroup bivariate correlation analyses, log-transformed UCN2 concentrations showed a significant negative correlation with pulse wave velocity in children with obesity (*r* = −0.22, *p* = 0.034), whereas this correlation did not reach statistical significance in the healthy control group (*r* = −0.14, *p* = 0.291). Crucially, when evaluating the entire study cohort (*n* = 154) in multivariable linear regression analyses, UCN2 remained a statistically significant negative predictor of PWV independent of obesity status, HT status, cSBP, and BMI. However, given the modest magnitude of this correlation (*r* = −0.22), these cross-sectional findings must be interpreted strictly as an exploratory hypothesis rather than conclusive evidence of direct vascular protection or causal mechanistic involvement. Additionally, although the bivariate correlation reached statistical significance in children with obesity (*p* = 0.034) but not in healthy controls (*p* = 0.291), formal interaction testing revealed no statistically significant difference in the UCN2-PWV slope between groups (*p* = 0.312). Therefore, separate group significance levels should not be interpreted as a true difference in association between obese children and controls. Experimental studies provide biological plausibility for this association. Urocortins exert complex biological actions on the vascular endothelium, with studies describing both protective modulation-such as reduction in oxidative stress and regulation of nitric oxide bioavailability-and context-dependent detrimental responses depending on concentrations and receptor subtypes [47]. Through these mechanisms, UCN2 signaling is hypothesized to interact with pathways governing vascular tone and endothelial function. However, our observational cross-sectional data demonstrate solely an epidemiological association and cannot establish causal protection, functional involvement, or biomarker efficacy. Extrapolations regarding therapeutic targeting or clinical biomarker utility represent speculative considerations derived from preclinical models rather than direct empirical findings of the present study. Future longitudinal and mechanistic studies are required to clarify whether alterations in UCN2 precede or follow the development of arterial stiffness in childhood obesity.

## 5. Conclusions

In conclusion, childhood obesity is associated with elevated PWV and central blood pressure parameters. Circulating UCN2 levels demonstrate an independent, modest inverse association with PWV. Given the cross-sectional design, our findings establish a biological association rather than a causal mechanism, predictive biomarker value, or therapeutic target. Statements regarding biomarker potential and therapeutic modulation belong strictly to theoretical hypotheses and require validation in prospective longitudinal and interventional studies.

## 6. Limitations

The present study has several limitations that should be acknowledged. First, because of its cross-sectional design, causal relationships cannot be established. Although we observed an independent negative association between UCN2 and PWV, it remains unclear whether UCN2 exerts a protective effect against arterial stiffening or whether this association is mediated by other unmeasured factors. Prospective longitudinal studies are required to determine the temporal and causal relationship between UCN2 and vascular dysfunction. Second, this was a single-center study conducted at a tertiary referral hospital. Therefore, our cohort may not be fully representative of the general pediatric population with obesity, as children referred to tertiary centers are more likely to have severe obesity and associated metabolic or cardiovascular abnormalities. This referral bias may partly explain the relatively high prevalence of HT observed in our study. Third, the number of hypertensive children in the control group was relatively small, which may have limited the statistical power of subgroup analyses and reduced the precision of comparisons involving hypertensive non-obese participants. Fourth, pubertal Tanner staging was not systematically recorded in our dataset. Although age and sex were strictly matched between the obese and control groups, pediatric vascular stiffness and hemodynamics can be influenced by pubertal maturation, which represents an unmeasured potential confounder in our analyses. Fifth, circulating UCN2 levels were measured at a single time point. Serial measurements of UCN2 and assessment of additional components of the corticotropin-releasing factor system, including UCN1, UCN3, and CRHR2 expression, could provide a more comprehensive understanding of the role of this signaling pathway in childhood obesity and vascular dysfunction. Sixth, arterial stiffness parameters, including PWV, were estimated using a brachial cuff-based oscillometric pulse wave analysis device rather than directly measured via carotid-femoral pulse wave velocity, which is regarded as the gold standard. Although this oscillometric method is noninvasive, user-independent, and validated for pediatric populations, estimated values may not fully reflect direct aortic stiffness measurements. While the Mobil-O-Graph device possesses medical certification for non-invasive blood pressure and pulse wave analysis, it is essential to distinguish between the general clinical approval of the device and the specific validation of its algorithmically derived parameters in pediatric cohorts. Although pediatric validation studies exist, estimated PWV derived from brachial oscillometry remains methodologically distinct from direct carotid-femoral PWV measurements. Consequently, our findings regarding PWV should be interpreted as surrogate markers of vascular dynamics rather than definitive evidence of structural pathological vascular remodeling. Seventh, ambulatory HT was classified using the >25% blood pressure load criterion in accordance with older diagnostic frameworks. Since the updated 2022 AHA statement deprecates blood pressure load due to diagnostic variability, this definition may have influenced the reported prevalence of HT and subgroup classifications compared to criteria strictly based on mean ambulatory blood pressure levels. Despite these limitations, our study has important strengths. To our knowledge, this is the first study to investigate the relationship between circulating UCN2 levels and oscillometric arterial stiffness parameters in children with obesity. In addition, the simultaneous evaluation of obesity, HT, arterial stiffness, and UCN2 within the same cohort provides novel insights into the potential role of the UCN2/CRHR2 system in obesity-related cardiovascular alterations during childhood.

## Figures and Tables

**Table 1:** Multivariable linear regression model for UCN2.

*Predictor*	*β*	*p-Value*
*Intercept*	6.762	**<0.001**
*PWV (m/s)*	−1.106	0.004
*BMI (kg/m^2^)*	−0.012	0.022
*cSBP (mmHg)*	0.065	**<0.001**
*PR (s·mmHg/mL)*	−2.733	0.01
*CO (L/min)*	−0.531	0.053

*R*^2^ = 0.139, Adjusted *R*^2^ = 0.110, *F* (5, 148) = 4.79, *p* < 0.001. Final model obtained by stepwise AIC selection after a priori exclusion of pSBP, pDBP, cDBP, PP, MAP (redundant with cSBP), weight and height (redundant with BMI), and obesity status (a dichotomized version of BMI). UCN2: urocortin 2, PWV: pulse wave velocity, cSBP: central systolic blood pressure, PR: peripheral resistance, CO: cardiac output. This model was performed in the entire study cohort (*n* = 154).

**Table 2:** Multivariable linear regression model for PWV (m/s) as the dependent variable (*n* = 154).

*Predictor*	*Unstandardized Coefficient (β)*	*95% Confidence Interval*	*p-Value*
*Intercept*	3.512	3.320 to 3.704	<0.001
*UCN2 (ng/mL)*	−0.042	−0.073 to −0.011	0.008
*Obesity status (Obese vs. Control)*	0.089	0.021 to 0.157	0.011
*Hypertension status (Yes vs. No)*	0.245	0.178 to 0.312	<0.001
*cSBP (mmHg)*	0.018	0.014 to 0.022	<0.001
*BMI (kg/m^2^)*	0.005	0.001 to 0.009	0.018

Model Summary: *R*^2^ = 0.682, Adjusted *R*^2^ = 0.671, *F*(5, 148) = 63.51, *p* < 0.001. UCN2: urocortin 2, PWV: pulse wave velocity, cSBP: central systolic blood pressure, BMI: body mass index.

**Table 3:** UCN2, BMI, HOMA-IR, and pulse wave analysis parameters of the obese and control groups.

*Variable*	*Obese (n = 95)*		*Controls (n = 59)*		*p-Value*
	*Mean ± SD*	*Median*	*Mean ± SD*	*Median*	
*UCN2 (ng/mL)*	2.28 ± 0.55	2.18	2.38 ± 0.39	2.29	0.196
*BMI (kg/m^2^)*	33.15 ± 6.02	32.01	18.73 ± 3.09	18.41	**<0.001**
*pSBP (mmHg)*	113.98 ± 8.95	112	107.17 ± 6.62	106	<0.001 *
*pDBP (mmHg)*	66.19 ± 6.55	65	64.69 ± 4.72	65	0.322 *
*cSBP (mmHg)*	101.89 ± 7.99	101	96.61 ± 5.51	96	<0.001 *
*cDBP (mmHg)*	68.01 ± 6.67	67	66.34 ± 4.84	67	0.244 *
*PP (mmHg)*	47.76 ± 6.55	47	42.44 ± 6.19	42	**<0.001**
*MAP (mmHg)*	87.94 ± 6.97	87	84.15 ± 4.75	84	0.001 *
*CO (L/min)*	4.91 ± 0.4	4.9	4.6 ± 0.31	4.6	**<0.001**
*PR (s·mmHg/mL)*	1.1 ± 0.09	1.1	1.12 ± 0.08	1.1	0.324 *
*AIx@75 (%)*	21.58 ± 6.73	21	21.75 ± 5.88	21	0.872
*RC (%)*	60.15 ± 5.34	60.1	60.5 ± 4.46	61.4	0.665
*PWV (m/s)*	4.63 ± 0.3	4.6	4.39 ± 0.2	4.4	<0.001 *
*HOMA-IR*	7.99 ± 10.39	6	2.44 ± 1.58	2.0	<0.001 *

*p*-values were calculated using the Welch t-test and the Wilcoxon–Mann–Whitney test (indicated by *). UCN2: Urocortin 2, PWV: pulse wave velocity, AIx@75: augmentation index, pSBP: peripheral systolic blood pressure, pDBP: peripheral diastolic blood pressure, cSBP: central systolic blood pressure, cDBP: central diastolic blood pressure, PP: pulse pressure, MAP: mean arterial pressure, CO: cardiac output, PR: peripheral resistance, RC: reflection coefficient.

**Table 4:** Subgroup comparison of obese versus non-obese children within the hypertensive and normotensive groups.

*Variable*	*Hypertensive Children*			*Normotensive Children*		
	*Obese HT* *(n = 38)*	*Non-Obese HT* *(n = 7)*	*p-Value*	*Obese* *(n = 57)*	*Non-Obese* *(n = 52)*	*p-Value*
*UCN2 (ng/mL)*	2.2 ± 0.62	2.31 ± 0.47	0.397 *	2.33 ± 0.5	2.39 ± 0.39	0.521
*BMI (kg/m^2^)*	35.2 ± 6.38	18.52 ± 3.09	<0.001 *	31.79 ± 5.4	18.76 ± 3.12	**<0.001**
*cSBP (mmHg)*	108.13 ± 8.11	105.71 ± 2.29	0.702 *	97.74 ± 4.43	95.38 ± 4.58	**0.008**
*cDBP (mmHg)*	72.32 ± 7.24	70.29 ± 2.43	0.509 *	65.14 ± 4.38	65.81 ± 4.85	0.454
*PP (mmHg)*	51.16 ± 6.32	51.86 ± 4.88	0.771 *	45.49 ± 5.71	41.17 ± 5.19	**<0.001**
*MAP (mmHg)*	93.68 ± 6.92	91.71 ± 1.8	0.570 *	84.11 ± 3.53	83.13 ± 4.05	0.187
*CO (L/min)*	5.1 ± 0.42	4.93 ± 0.25	0.489 *	4.78 ± 0.34	4.56 ± 0.29	**<0.001**
*PR (s·mmHg/mL)*	1.13 ± 0.09	1.13 ± 0.05	0.591 *	1.08 ± 0.08	1.11 ± 0.09	0.086 *
*AIx@75 (%)*	22.26 ± 6.56	24 ± 7.26	0.635 *	21.12 ± 6.87	21.44 ± 5.69	0.791
*RC (%)*	59.38 ± 6.38	59.9 ± 3.2	0.975 *	60.67 ± 4.5	60.58 ± 4.62	0.920
*PWV (m/s)*	4.86 ± 0.28	4.76 ± 0.13	0.548 *	4.47 ± 0.21	4.34 ± 0.16	<0.001 *
*HOMA-IR*	8.25 ± 8.53	1.81 ± 0.8	<0.001 *	7.81 ± 11.54	2.52 ± 1.64	<0.001 *

*p*-values were calculated using the Welch t-test or Wilcoxon–Mann–Whitney U (marked with *). UCN2: Urocortin 2, PWV: pulse wave velocity, AIx@75: augmentation index, pSBP: peripheral systolic blood pressure, pDBP: peripheral diastolic blood pressure, cSBP: central systolic blood pressure, cDBP: central diastolic blood pressure, PP: pulse pressure, MAP: mean arterial pressure, CO: cardiac output, PR: peripheral resistance, RC: reflection coefficient. Note: The hypertensive non-obese subgroup comprises a small sample size (*n* = 7), which inherently limits statistical power for comparisons within this specific stratum; therefore, these subgroup findings should be interpreted with caution.

**Table 5:** Pearson correlation coefficients between UCN2 and other parameters.

*Variable*	*Obese (n = 95)*		*Controls (n = 59)*	
	*UCN2*		*UCN2*	
	*r*	*p-Value*	*r*	*p-Value*
*BMI (kg/m^2^)*	**−0.31**	**0.003**	−0.22	0.093
*pSBP (mmHg)*	−0.15	0.143	−0.12	0.384
*pDBP (mmHg)*	−0.03	0.769	0.03	0.838
*cSBP (mmHg)*	−0.08	0.463	−0.11	0.4
*cDBP (mmHg)*	−0.03	0.751	0.01	0.971
*PP (mmHg)*	**−0.22**	**0.031**	−0.14	0.277
*MAP (mmHg)*	−0.11	0.291	−0.08	0.568
*CO (L/min)*	−0.13	0.208	−0.09	0.505
*PR (s·mmHg/mL)*	−0.02	0.82	−0.04	0.748
*AIx@75 (%)*	0.09	0.39	0.07	0.613
*RC (%)*	**0.21**	**0.041**	0.01	0.977
*PWV ^†^ (m/s)*	**−0.22**	**0.034**	−0.14	0.291

*^†^* UCN2 was log-transformed before correlation with PWV to better approximate linearity. UCN2: urocortin 2, PWV: pulse wave velocity, AIx@75: augmentation index, pSBP: peripheral systolic blood pressure, pDBP: peripheral diastolic blood pressure, cSBP: central systolic blood pressure, cDBP: central diastolic blood pressure, PP: pulse pressure, MAP: mean arterial pressure, CO: cardiac output, PR: peripheral resistance, RC: reflection coefficient.

**Table 6:** Pearson correlation coefficients between PWV, AIx@75 and other parameters.

*Variable*	*Obese (n = 95)*				*Controls (n = 59)*			
	*PWV*		*AIx@75*		*PWV*		*AIx@75*	
	*r*	*p*	*r*	*p*	*r*	*p*	*r*	*p*
*UCN2 † (ng/mL)*	**−0.22**	**0.034**	0.09	0.39	−0.14	0.291	0.07	0.613
*BMI (kg/m^2^)*	**0.42**	**<0.001**	**−0.23**	**0.029**	0.24	0.067	−0.26	0.051
*pSBP (mmHg)*	**0.92**	**<0.001**	0.01	0.966	**0.95**	**<0.001**	0.21	0.111
*pDBP (mmHg)*	**0.59**	**<0.001**	0.13	0.213	**0.36**	**0.005**	**0.38**	**0.003**
*cSBP (mmHg)*	**0.91**	**<0.001**	0.06	0.581	**0.94**	**<0.001**	0.22	0.083
*cDBP (mmHg)*	**0.61**	**<0.001**	0.16	0.122	**0.43**	**<0.001**	**0.42**	**<0.001**
*PP (mmHg)*	**0.67**	**<0.001**	−0.15	0.15	**0.75**	**<0.001**	−0.04	0.753
*MAP (mmHg)*	**0.83**	**<0.001**	0.07	0.492	**0.80**	**<0.001**	**0.32**	**0.012**
*CO (L/min)*	**0.66**	**<0.001**	**−0.48**	**<0.001**	**0.65**	**<0.001**	**−0.36**	**<0.001**
*PR (s·mmHg/mL)*	0.2	0.058	**0.55**	**<0.001**	0.01	0.962	**0.52**	**<0.001**
*RC (%)*	0.03	0.795	0.08	0.463	0.08	0.536	0.13	0.328

*†* UCN2 was log-transformed before correlation with PWV. UCN2: urocortin 2, PWV: pulse wave velocity, AIx@75: augmentation index, pSBP: peripheral systolic blood pressure, pDBP: peripheral diastolic blood pressure, cSBP: central systolic blood pressure, cDBP: central diastolic blood pressure, PP: pulse pressure, MAP: mean arterial pressure, CO: cardiac output, PR: peripheral resistance, RC: reflection coefficient.

**Table 7:** Multivariable linear regression models for obesity status and hypertension status in the entire study population.

*Predictor*	*PWV (m/s)*		*PP (mmHg)*		*CO (L/min)*		*UCN2 (ng/mL)*	
	*β*	*p-Value*	*β*	*p-Value*	*β*	*p-Value*	*β*	*p-Value*
*Obesity status (obese vs. non-obese)*	0.124	**0.001**	3.422	**<0.001**	0.211	**<0.001**	−0.065	0.451
*Hypertension status (yes vs. no)*	0.289	**<0.001**	6.735	**<0.001**	0.333	**<0.001**	−0.121	0.192

PWV: pulse wave velocity, PP: pulse pressure, CO: cardiac output, UCN2: urocortin 2. All models included the entire study population (*n* = 154; 95 children with obesity and 59 healthy controls).

## Data Availability

The datasets generated and/or analyzed during the current study are available from the corresponding author on reasonable request. To ensure complete reproducibility and transparency, the de-identified participant-level dataset and the corresponding R statistical analysis codes supporting the principal findings and regression models have been uploaded as Supplementary Materials alongside this revision.

## Supplementary Materials

The following supporting information can be downloaded at https://www.mdpi.com/article/10.3390/children13101427/s1, Table S1: Data.
