# Supplementary material for: The Role of Urocortin 2 in Arterial Stiffness and Hypertension in Children with Obesity
Source: Children (Basel). 2026 Oct 18;13(10):1427. doi: 10.3390/children13101427 (PMC13702873; doi:10.3390/children13101427)
Supplement: Supplementary file 1 [file children-13-01427-s001.zip › children-4580188-supplementary.pdf]

	No.	Urocortin	Age	Sex	Weight	Height	BMI
HST0001	1	1,621	14,20	Male	103,00	157,00	41,86
HST0002	2	1,931	17,75	Male	101,00	170,00	34,95
HST0003	3	1,803	13,42	Female	87,00	159,00	34,41
HST0004	4	1,525	12,50	Male	99,50	165,00	36,50
HST0005	5	3,024	17,55	Male	115,00	183,00	34,34
HST0006	6	3,242	9,85	Male	56,00	137,00	29,84
HST0007	7	2,707	10,62	Male	52,00	145,00	24,73
HST0008	8	2,394	14,00	Male	128,50	161,50	49,27
HST0009	9	1,999	16,94	Female	88,50	174,00	29,23
HST0010	10	1,995	10,84	Female	71,00	144,00	34,20
HST0011	11	2,151	10,00	Male	56,00	150,50	24,72
HST0012	12	2,014	15,10	Female	100,00	155,00	41,62
HST0013	13	4,027	11,00	Male	71,00	157,50	28,62
HST0014	14	4,319	11,50	Male	63,60	153,00	27,10
HST0015	15	1,124	16,70	Male	127,00	173.5	42,19
HST0016	16	1,825	14,12	Male	108,00	173,50	35,88
HST0017	17	2,667	14,44	Female	105,50	170,00	36,51
HST0018	18	2,701	9,20	Male	51,00	132,00	29,27
HST0019	19	3,508	10,68	Female	56,00	154,00	23,61
HST0020	20	2,288	14,20	Female	98,50	162,00	37,53
HST0021	21	2,353	15,32	Female	92,50	162,50	35,03
HST0022	22	2,472	12,42	Male	70,50	160,00	27,54
HST0023	23	2,254	13,81	Female	95,00	161,50	36,42
HST0024	24	1,845	15,84	Male	127,00	177,00	40,54
HST0025	25	2,225	9,83	Male	58,90	148,00	26,89
HST0026	26	2,986	10,18	Male	48,50	142,50	23,88
HST0027	27	2,598	12,77	Female	75,00	159,50	29,48
HST0028	28	2,550	17,56	Male	129,00	184,00	38,10
HST0029	29	2,397	15,56	Female	100,50	165,50	36,69
HST0030	30	1,777	11,85	Male	91,10	155,00	37,92
HST0031	31	2,607	16,2	Female	80,00	162,00	30,50
HST0032	32	2,648	9,30	Female	60,30	139,00	31,21
HST0033	33	3,052	11,12	Male	53,00	142,00	26,28
HST0034	34	1,522	13,92	Female	94,00	172,00	31,77
HST0035	35	2,107	12,10	Female	66,60	154,00	28,08
HST0036	36	2,427	11,58	Female	72,50	147,00	33,55
HST0037	37	2,974	14,54	Female	81,70	163,00	30,75
HST0038	38	1,483	14,60	Male	83,00	172,00	28,06
HST0039	39	1,811	11,95	Male	91,00	152,00	39,39
HST0040	40	1,860	10,15	Female	67,00	131,50	38,75
HST0041	41	1,700	9,56	Male	51.8	143,50	25,16
HST0042	42	2,628	9,57	Female	54,00	143,00	26.41
HST0043	43	2,978	11,93	Male	76,50	160,00	29,88
HST0044	44	2,186	15,00	Female	106,00	159,00	41,93
HST0045	45	1,982	16,10	Female	144,00	182,00	43,47
HST0046	46	2,053	9,10	Female	45,00	136,00	24,33
HST0047	47	2,024	15,48	Male	96,90	174,00	32,01
HST0048	48	2,163	12,50	Male	106,00	160,00	41,41
HST0049	49	2,161	13,50	Female	84,00	168,00	29,76
HST0050	50	1,989	10,37	Female	94,00	135,00	34,53
HST0051	51	1,945	13,20	Male	83,50	155,00	34,86
HST0052	52	2,144	10,00	Male	53,50	140,50	27,10

HST0053	53	2,472	15,78	Male	88,70	174,00	29,30
HST0054	54	2,403	14,41	Female	79,20	157,00	32,10
HST0055	55	1,775	12,43	Male	87,00	162,00	33,15
HST0056	56	2,184	17,50	Male	119,00	180,00	36,73
HST0057	57	2,663	10,30	Male	64,50	149,00	29,05
HST0058	58	2,652	10,30	Female	59,00	153,00	25,20
HST0059	59	1,534	14,90	Male	133,00	168,00	47,12
HST0060	60	1,651	14,45	Male	70,80	159,00	28,01
HST0061	61	2,408	10,33	Male	61,30	140,00	31,28
HST0062	62	2,575	10,23	Male	61,50	148,00	28,08
HST0063	63	2,178	16,58	Male	114,00	181,00	34,80
HST0064	64	1,711	15,40	Male	162,00	179,00	50,06
HST0065	65	2,616	12,75	Male	78,30	156,00	32,10
HST0066	66	2,045	12,16	Male	118,50	170,00	41,00
HST0067	67	2,532	15,41	Male	72,80	160,00	28,44
HST0068	68	2,150	17,15	Male	110,00	180,00	35,95
HST0069	69	3,266	16,50	Female	102,00	173,00	34,02
HST0070	70	3,006	10,35	Female	64,00	147,00	29,62
HST0071	71	2,215	14,45	Male	126,40	168,70	44,40
HST0072	72	1,695	14,25	Male	100,50	167,00	38,83
HST0073	73	3,576	14,00	Female	85,00	150,00	37,78
HST0074	74	2,660	16,02	Female	126,00	167,00	45,18
HST0075	75	2,022	14,00	Female	85,00	165,00	31,22
HST0076	76	2,901	10,20	Male	60,60	145,00	28,82
HST0077	77	2,440	14,00	Male	85,00	173,00	28,40
HST0078	78	2,075	14,25	Male	82,00	162,00	31,25
HST0079	79	2,186	14,25	Female	81,00	147,00	37,48
HST0080	80	2,086	12,10	Male	81,20	154,50	34,02
HASTA81	81	2,699	14,25	Male	104,50	165,00	38,38
HASTA82	82	2,103	16,02	Male	110,00	173,00	36,75
HASTA83	83	2,129	9,22	Male	48,00	147,00	26,84
HASTA84	84	2,196	11,66	Male	82,00	170,00	28,37
HASTA85	85	2,053	14,33	Male	113,00	172,60	37,93
HASTA86	86	1,276	17,86	Male	89,00	171,00	30,44
HASTA87	87	1,541	10,00	Male	61,00	145,00	29,01
HASTA88	88	1,968	12,57	Female	61,00	153,00	26,06
HASTA89	89	1,907	17,13	Female	84,00	163,00	31,60
HASTA90	90	2,223	16,87	Male	100,00	161,00	38,58
HASTA91	91	1,845	16,09	Male	110,00	178,00	34,72
HASTA92	92	2,062	11,48	Male	69,70	149,00	31,39
HASTA94	94	2,725	12,68	Male	70,00	160,00	27,34
HASTA95	95	2,213	10,72	Male	55,00	151,00	24,12
HASTA96	96	1,971	12,00	Female	57,00	142,50	28,00
HASTA97	97	1,897	13,14	Male	86,00	172,00	29,07
KONT0001	1	1,699	13,25	Female	55,00	166,00	19,96
KONT0002	2	2,116	13,50	Male	46,00	159,00	18,20
KONT0004	4	3,272	12,00	Male	56,00	158,00	22,43
KONT0005	5	2,097	17,50	Male	63,00	178,00	19,88
KONT0006	6	1,857	11,90	Male	41,40	148,30	18,82
KONT0007	7	2,389	10,52	Male	37,00	148,00	16,89
KONT0008	8	1,848	17,00	Female	63,00	168,00	22,32
KONT0009	9	2,584	14,45	Female	47,30	165,00	17,37
KONT0010	10	1,912	17,00	Male	54,00	160,00	21,09
KONT0011	11	2,382	12,66	Female	36,00	155,00	14,98

KONT0012	12	2,183	12,70	Female	53,00	164,00	19,75
KONT0013	13	2,037	14,25	Male	54,00	162,00	20,58
KONT0014	14	2,724	13,25	Female	36,00	156,00	14,79
KONT0015	15	3,034	12,11	Male	32,00	142,00	15,79
KONT0016	16	2,079	10,50	Male	34,00	143,00	16,63
KONT0017	17	2,845	9,58	Female	39,00	137,00	20,78
KONT0018	18	2,326	17,00	Female	46,00	165,00	16,40
KONT0019	19	1,979	12,83	Female	55,00	165,00	20,20
KONT0020	20	1,960	11,65	Male	37,20	142,00	18,45
KONT0021	21	2,633	14,07	Female	51,00	167,00	18,29
KONT0022	22	2,679	11,73	Female	50,50	158,50	20,10
KONT0023	23	2,062	11,75	Male	44,00	150,00	19,56
KONT0024	24	2,387	14,61	Female	39,00	157,00	15,82
KONT0025	25	2,223	17,50	Female	48,00	170,00	16,60
KONT0027	27	3,240	9,91	Male	37,00	144,50	17,72
KONT0028	28	2,565	15,47	Male	41,50	159,50	16,31
KONT0029	29	2,285	17,50	Female	71,40	170,00	24,71
KONT0030	30	1,920	13,08	Male	41,90	147,50	19,26
KONT0031	31	2,169	12,16	Female	57,30	164,00	21,30
KONT0032	32	2,769	10,08	Male	30,60	144,50	14,65
KONT0033	33	2,832	11,75	Female	29,70	143,00	14,52
KONT0034	34	2,243	13,75	Male	55,00	173,00	18,38
KONT0035	35	2,028	13,75	Female	45,70	169,00	16,00
KONT0036	36	2,495	14,90	Female	45,30	158,30	18,08
KONT0037	37	1,846	10,02	Male	26,70	136,00	14,44
KONT0038	38	2,046	12,64	Male	43,50	150,00	19,33
KONT0039	39	2,354	9,95	Female	28,40	131,00	16,55
KONT0040	40	2,574	11,33	Male	38,50	143,00	18,83
KONT0041	41	2,801	14,69	Male	69,30	170,00	23,98
KONT0042	42	2,904	13,30	Female	49,50	164,00	18,40
KONT0043	43	2,278	17,03	Male	68,80	184,00	20,32
KONT0044	44	2,256	16,91	Male	65,80	176,00	21,24
KONT0045	45	3,636	12,91	Male	31,70	157,00	12,86
KONT0046	46	2,242	11,04	Male	43,00	150,50	18,98
KONT0047	47	2,286	17,32	Female	50,00	163,00	18,32
KONT0048	48	2,107	11,71	Male	48,40	150,00	21,51
KONT0049	49	2,549	11,10	Male	31,10	140,00	15,86
KONT0050	50	2,695	10,00	Male	27,00	140,00	13,78
KONT0051	51	2,313	14,39	Male	68,00	168,00	24,09
KONT0052	52	1,868	15,33	Male	67,00	170,00	23,18
KONT0053	53	2,846	10,01	Male	36,00	132,50	20,51
KONT0054	54	2,279	14,20	Male	41,00	150,00	18,41
KONT0055	55	2,343	13,23	Male	49,50	165,00	18,18
KONT0056	56	2,391	16,70	Male	91,00	185,00	26,59
KONT0057	57	2,277	16,63	Male	71,00	165,00	26,08
KONT0058	58	2,801	11,02	Female	47,00	149,00	21,17
KONT0059	59	2,520	11,85	Male	39,00	158,00	15,62

KONT0060	60	2,132	9,55	Male	29,40	140,00	15,00
KONT0061	61	2,195	11,24	Female	34,00	150,00	15,11

Control no. 3 was excluded.
Control no. 26 was included in the patient group.

Hasta grubunda 93 nolu ha
Kontrol grubunda 59 olgunu

SDS	Peripheral Systolic BP / Peripheral Diastolic BP / Central Systolic BP / Central Diastolic BP				
3,62	110/95	116,00	62,00	100,00	60,00
2,53	115/90	123,00	65,00	106,00	67,00
3,28	105/77	111,00	56,00	101,00	59,00
3,13	127/97	115,00	68,00	100,00	69,00
2,47	150/100	128,00	79,00	118,00	80,00
2,80	105/72	104,00	62,00	93,00	64,00
1,83	110/85	115,00	64,00	99,00	66,00
4,25	123/80	115,00	61,00	101,00	64,00
2,37	128/107	111,00	67,00	99,00	68,00
3,26	135/64	108,00	58,00	97,00	59,00
2,03	110/80	105,00	65,00	99,00	68,00
4,13	120/78	117,00	68,00	104,00	71,00
2,30	125/70	108,00	64,00	94,00	65,00
2,00	109/70	125,00	68,00	115,00	69,00
3,37	130/75	133,00	71,00	118,00	72,00
3,01	125/80	115,00	65,00	104,00	66,00
3,57	120/80	113,00	72,00	102,00	74,00
2,94	135/75	115,00	69,00	103,00	71,00
1,70	165/100	117,00	63,00	104,00	65,00
3,70	105/75	107,00	62,00	100,00	65,00
3,37	120/90	110,00	69,00	98,00	71,00
1,89	120/75	103,00	61,00	94,00	62,00
3,55	115/80	117,00	71,00	105,00	70,00
3,30	135/80	117,00	71,00	109,00	72,00
2,45	110/80	116,00	66,00	101,00	68,00
1,85	100/70	103,00	61,00	93,00	63,00
2,51	90/55	109,00	63,00	95,00	65,00
2,92	160/100	142,00	97,00	132,00	99,00
3,58	110/70	111,00	58,00	103,00	60,00
3,24	135/85	113,00	64,00	103,00	67,00
2,64	125/90	The device was not fitted.			
3,07	125/65	106,00	61,00	97,00	64,00
1,93	108/70	111,00	62,00	101,00	65,00
2,88	130/70	110,00	56,00	96,00	58,00
2,31	105/70	96,00	59,00	89,00	60,00
3,16	100/60	112,00	59,00	97,00	61,00
2,70	120/65	116,00	63,00	105,00	67,00
1,78	150/80	117,00	60,00	102,00	61,00
3,38	135/80	113,00	74,00	102,00	76,00
3,65	100/65	101,00	68,00	91,00	70,00
2,27	100/80	102,00	60,00	89,00	60,00
2,44	120/85	104,00	66,00	94,00	65,00
2,30	115/60	102,00	63,00	93,00	63,00
4,17	125/65	105,00	67,00	95,00	69,00
3,52	154/80	146,00	82,00	129,00	86,00
2,30	115/50	104,00	64,00	93,00	67,00
2,35	125/80	108,00	62,00	92,00	60,00
3,62	120/87	128,00	68,00	112,00	72,00
2,53	140/80	109,00	59,00	95,00	59,00
3,31	130/90	117,00	68,00	101,00	71,00
2,93	130/80	130,00	73,00	114,00	75,00
2,44	125/80	109,00	69,00	100,00	68,00

1,87	130/75	107,00	65,00	100,00	66,00
2,94	100/65	96,00	53,00	85,00	54,00
2,73	125/70	116,00	68,00	103,00	70,00
2,77	155/70	121,00	68,00	107,00	68,00
2,55	150/60	115,00	68,00	104,00	71,00
2,08	120/75	106,00	65,00	96,00	67,00
4,15	140/80	130,00	77,00	116,00	79,00
1,96	105/60	107,00	63,00	98,00	64,00
2,79	145/93	120,00	76,00	108,00	79,00
2,44	130/80	107,00	62,00	94,00	63,00
2,62	130/60	112,00	59,00	99,00	63,00
4,06	140/80	133,00	76,00	117,00	76,00
2,59	120/65	110,00	64,00	99,00	66,00
3,54	130/85	119,00	52,00	102,00	55,00
1,97	105/75	111,00	66,00	100,00	69,00
2,46	120/65	109,00	59,00	93,00	62,00
3,22	120/70	110,00	67,00	100,00	68,00
2,86	100/75	118,00	76,00	106,00	77,00
3,81	110/90	127,00	77,00	113,00	80,00
3,21	145/75	124,00	70,00	111,00	73,00
3,73	120/70	112,00	64,00	102,00	65,00
4,36	130/95	103,00	64,00	96,00	66,00
2,78	130/85	122,00	74,00	107,00	76,00
2,85	100/70	111,00	60,00	95,00	62,00
1,91	95/60	110,00	59,00	100,00	61,00
2,36	130/80	124,00	72,00	110,00	73,00
3,64	140/90	128,00	73,00	118,00	76,00
2,83	140/85	115,00	70,00	105,00	72,00
3,27	120/70	112,00	66,00	102,00	69,00
2,91	110/55	111,00	64,00	99,00	69,00
2,65	120/85	111,00	72,00	98,00	74,00
2,13	140/60	123,00	64,00	104,00	68,00
3,22	130/80	111,00	67,00	101,00	69,00
1,84	105/65	114,00	62,00	94,00	66,00
2,96	115/65	112,00	61,00	99,00	63,00
1,86	120/70	117,00	63,00	101,00	65,00
2,83	120/70	117,00	72,00	108,00	74,00
3,02	125/70	110,00	65,00	102,00	66,00
2,66	130/80	119,00	70,00	108,00	70,00
2,58	110/70	107,00	62,00	97,00	65,00
1,83	105/70	123,00	69,00	106,00	70,00
1,68	110/80	108,00	72,00	98,00	74,00
2,39	140/90	104,00	65,00	96,00	66,00
2,11	130/85	118,00	74,00	106,00	76,00
	130/80	120,00	71,00	108,00	73,00
	115/90	116,00	68,00	105,00	69,00
	90/65	105,00	69,00	96,00	70,00
	125/100	123,00	70,00	108,00	72,00
	100/60	100,00	57,00	91,00	59,00
	110/60	106,00	60,00	93,00	63,00
	120/70	106,00	60,00	95,00	60,00
	110/90	105,00	67,00	95,00	68,00
	110/80	110,00	75,00	100,00	77,00
	130/90	117,00	69,00	103,00	71,00

110/70	108,00	60,00	94,00	61,00
120/85	107,00	74,00	99,00	76,00
100/65	105,00	68,00	95,00	69,00
115/90	119,00	65,00	105,00	67,00
100/65	106,00	66,00	93,00	68,00
110/65	99,00	56,00	89,00	58,00
110/75	99,00	65,00	91,00	66,00
100/55	101,00	62,00	89,00	64,00
135/95	123,00	67,00	105,00	70,00
120/75	109,00	67,00	99,00	70,00
110/70	108,00	63,00	99,00	66,00
100/50	108,00	67,00	100,00	68,00
85/65	104,00	62,00	94,00	64,00
90/50	113,00	68,00	103,00	72,00
105/70	105,00	69,00	95,00	69,00
120/80	106,00	70,00	97,00	70,00
110/70	99,00	60,00	93,00	62,00
115/90	102,00	64,00	94,00	65,00
100/75	102,00	54,00	91,00	55,00
100/70	99,00	62,00	84,00	61,00
115/80	105,00	67,00	97,00	69,00
125/75	105,00	66,00	99,00	67,00
125/80	105,00	65,00	98,00	66,00
110/80	95,00	59,00	89,00	61,00
120/65	108,00	66,00	98,00	69,00
100/75	109,00	63,00	100,00	66,00
95/65	102,00	70,00	93,00	71,00
90/65	105,00	63,00	96,00	65,00
130/70	123,00	65,00	108,00	67,00
110/75	102,00	63,00	95,00	64,00
105/70	108,00	63,00	100,00	64,00
110/80	109,00	66,00	100,00	69,00
120/70	109,00	69,00	99,00	71,00
110/60	108,00	63,00	95,00	65,00
120/80	115,00	72,00	103,00	74,00
105/65	105,00	59,00	95,00	59,00
110/80	108,00	68,00	96,00	68,00
95/70	92,00	57,00	83,00	58,00
125/65	104,00	53,00	92,00	55,00
125/65	106,00	64,00	93,00	65,00
110/70	102,00	61,00	91,00	63,00
100/60	110,00	59,00	95,00	62,00
115/70	115,00	70,00	103,00	72,00
125/60	111,00	62,00	102,00	63,00
125/75	114,00	60,00	101,00	62,00
120/80	112,00	66,00	100,00	69,00
110/70	103,00	68,00	92,00	69,00

110/80	104,00	68,00	94,00	69,00
100/80	99,00	67,00	90,00	69,00

Patient was excluded, Numara 92 The numbering proceeds from 92 to 94. One of the remaining 96 patients did not wear the device. Total: 95.

36 erkek 23 were female

Peripheral Pressure /	Central Aortic Pressure	Cardiac Output	Peripheral Resistance	Augmentation Index
54,00	87,00	5,10	1,00	21,00
58,00	91,00	5,40	1,10	20,00
55,00	81,00	3,90	1,30	35,00
47,00	89,00	4,90	1,10	17,00
49,00	101,00	4,80	1,20	18,00
40,00	83,00	4,70	1,00	18,00
51,00	87,00	4,80	1,10	13,00
53,00	86,00	5,40	1,00	12,00
44,00	87,00	4,90	1,10	18,00
50,00	81,00	4,30	1,20	27,00
40,00	83,00	4,50	1,20	22,00
49,00	90,00	4,80	1,10	21,00
44,00	84,00	4,90	1,00	22,00
57,00	94,00	4,90	1,20	29,00
63,00	99,00	4,80	1,30	20,00
50,00	88,00	5,30	1,00	14,00
41,00	85,00	5,10	1,10	19,00
46,00	90,00	4,70	1,20	24,00
46,00	88,00	4,60	1,20	32,00
45,00	83,00	4,90	1,10	13,00
40,00	88,00	4,80	1,10	33,00
42,00	80,00	4,60	1,10	21,00
47,00	90,00	5,10	1,10	28,00
46,00	92,00	5,30	1,10	19,00
50,00	89,00	4,80	1,10	28,00
42,00	80,00	4,40	1,10	27,00
46,00	84,00	4,60	1,10	23,00
45,00	118,00	5,70	1,30	27,00
53,00	82,00	4,80	1,10	28,00
49,00	87,00	4,40	1,20	24,00
45,00	82,00	4,60	1,10	27,00
49,00	84,00	5,30	1,00	14,00
54,00	81,00	4,90	1,00	25,00
37,00	76,00	4,30	1,10	17,00
53,00	83,00	4,60	1,10	21,00
53,00	87,00	5,10	1,10	25,00
58,00	86,00	4,70	1,10	20,00
39,00	92,00	4,70	1,20	20,00
34,00	83,00	4,20	1,20	37,00
42,00	79,00	4,80	1,00	16,00
38,00	83,00	4,30	1,10	30,00
39,00	81,00	4,40	1,10	19,00
37,00	84,00	4,40	1,20	28,00
63,00	111,00	6,10	1,10	17,00
40,00	83,00	4,20	1,20	33,00
46,00	83,00	5,20	0,90	5,00
60,00	95,00	5,10	1,20	13,00
51,00	82,00	4,60	1,10	32,00
49,00	90,00	5,10	1,10	25,00
57,00	99,00	5,40	1,10	27,00
41,00	87,00	4,80	1,10	17,00

42,00	84,00	4,90	1,00	21,00
43,00	73,00	4,50	1,00	11,00
48,00	90,00	5,40	1,00	20,00
53,00	92,00	5,20	1,10	11,00
47,00	89,00	5,00	1,10	33,00
41,00	84,00	4,40	1,10	33,00
53,00	101,00	5,50	1,10	30,00
45,00	83,00	4,80	1,10	19,00
44,00	96,00	4,50	1,30	30,00
45,00	82,00	4,90	1,00	18,00
53,00	83,00	4,70	1,10	24,00
58,00	102,00	6,10	1,00	12,00
46,00	85,00	4,80	1,10	23,00
67,00	82,00	5,10	1,00	18,00
45,00	86,00	4,90	1,10	24,00
50,00	82,00	5,10	1,00	12,00
43,00	86,00	5,20	1,00	14,00
42,00	95,00	4,40	1,30	27,00
50,00	99,00	5,70	1,10	22,00
54,00	95,00	4,90	1,20	24,00
48,00	86,00	5,10	1,00	18,00
40,00	82,00	4,90	1,00	14,00
48,00	96,00	5,10	1,10	28,00
52,00	83,00	4,90	1,00	22,00
51,00	83,00	4,60	1,10	25,00
52,00	96,00	5,30	1,10	20,00
55,00	98,00	5,10	1,20	33,00
45,00	91,00	4,80	1,10	26,00
46,00	87,00	4,70	1,20	20,00
47,00	86,00	4,90	1,10	17,00
38,00	90,00	4,60	1,20	28,00
59,00	91,00	5,60	1,00	17,00
45,00	87,00	5,10	1,10	13,00
52,00	86,00	5,20	1,00	17,00
51,00	84,00	4,80	1,10	21,00
54,00	88,00	5,20	1,00	22,00
45,00	93,00	4,80	1,20	26,00
45,00	86,00	5,50	1,00	4,00
50,00	92,00	5,70	1,00	8,00
45,00	83,00	5,00	1,00	18,00
54,00	94,00	5,30	1,10	17,00
35,00	88,00	4,50	1,20	23,00
39,00	83,00	4,50	1,10	27,00
45,00	94,00	4,90	1,20	19,00
49,00	93,00	5,10	1,10	21,00
48,00	90,00	4,60	1,20	19,00
36,00	85,00	4,50	1,10	21,00
52,00	94,00	5,30	1,10	13,00
42,00	77,00	4,40	1,10	18,00
46,00	81,00	4,80	1,00	22,00
46,00	81,00	4,30	1,20	18,00
37,00	84,00	4,30	1,20	28,00
35,00	91,00	4,40	1,20	24,00
48,00	91,00	4,90	1,10	36,00

48,00	82,00	4,80	1,00	18,00
34,00	89,00	4,20	1,30	23,00
37,00	85,00	4,00	1,30	33,00
54,00	90,00	4,50	1,20	27,00
40,00	84,00	4,20	1,20	31,00
43,00	76,00	4,20	1,10	28,00
35,00	81,00	4,40	1,10	21,00
39,00	80,00	4,40	1,10	21,00
56,00	93,00	4,80	1,20	28,00
42,00	86,00	4,90	1,10	23,00
45,00	83,00	4,50	1,10	34,00
41,00	87,00	4,70	1,10	20,00
42,00	81,00	4,90	1,00	17,00
45,00	89,00	4,90	1,10	23,00
36,00	85,00	4,80	1,10	14,00
36,00	86,00	4,40	1,20	23,00
38,00	78,00	4,30	1,10	20,00
38,00	81,00	4,20	1,20	21,00
48,00	76,00	4,50	1,00	21,00
36,00	79,00	4,40	1,10	15,00
38,00	84,00	4,40	1,20	35,00
39,00	84,00	5,00	1,00	18,00
41,00	83,00	4,60	1,10	28,00
36,00	76,00	4,40	1,10	17,00
42,00	85,00	4,60	1,10	26,00
46,00	84,00	5,00	1,10	21,00
31,00	85,00	4,20	1,20	24,00
42,00	82,00	4,90	1,00	20,00
59,00	92,00	5,00	1,10	20,00
39,00	81,00	4,90	1,00	16,00
45,00	84,00	5,00	1,00	10,00
42,00	86,00	4,80	1,10	18,00
40,00	87,00	4,40	1,20	20,00
43,00	84,00	5,00	1,00	13,00
44,00	92,00	4,60	1,20	32,00
46,00	80,00	4,60	1,10	17,00
40,00	86,00	4,40	1,20	23,00
35,00	73,00	4,30	1,00	9,00
51,00	77,00	4,80	1,00	13,00
42,00	83,00	4,60	1,10	19,00
41,00	79,00	4,60	1,00	24,00
51,00	82,00	5,20	1,00	17,00
46,00	91,00	4,90	1,10	25,00
49,00	84,00	4,50	1,20	22,00
54,00	85,00	4,80	1,10	18,00
46,00	87,00	4,60	1,10	27,00
35,00	84,00	4,60	1,10	23,00

37,00	85,00	4,40	1,20	21,00
32,00	82,00	3,80	1,30	26,00

Remaining patients: 61 male and 34 female

Reflection Coefficient	PWV	HOMA-IR / Hypertension		Stage 1 HT	Stage 2 HT	Elevated BP
56,40	4,60	55,10	Yes	Yes	No	No
55,70	4,90	6,60	No	No	No	Yes
70,90	4,50	8,66	No	No	No	No
51,90	4,60	7,60	Yes	Yes	No	No
64,30	5,10	6,42	Yes	Yes	No	No
58,90	4,30	1,80	No	No	No	No
61,10	4,50	2,86	Yes	Yes	No	No
59,60	4,60	5,08	No	No	No	
61,00	4,50	9,70	No	No	No	
64,70	4,40	4,68	No	No	No	
70,00	4,40	5,81	No	No	No	
59,20	4,70	4,20	Yes	Yes	No	No
51,70	4,40	7,54	No	No	No	
71,10	5,00	4,72	Yes	No	Yes	No
66,80	5,20	3,93	Yes	No	Yes	No
65,80	5,30	9,00	No	No	No	
59,80	4,50	3,99	No	No	No	
59,10	4,60	6,00	Yes	Yes	No	No
65,20	4,70	11,40	Yes	Yes	No	No
65,20	4,40	4,00	No	No	No	
49,80	4,50	6,70	Yes	Yes	No	No
64,40	4,30	4,00	No	No	No	
62,30	4,70	6,80	No	No	No	
59,00	4,80	9,40	Yes	Yes	No	No
59,20	4,60	4,90	Yes	No	Yes	No
61,50	4,30	10,30	No	No	No	
50,90	4,40	6,70	No	No	No	
62,80	5,50	6,80	Yes	No	Yes	No
66,70	4,70	2,60	No	No	No	
65,40	4,60	6,90	Yes	Yes	No	No
		3,60				
64,80	4,40	17,40	No	No	No	
66,00	4,50	1,80	No	No	No	
54,70	4,90	7,10	No	No	No	
63,30	4,30	3,90	No	No	No	
62,20	4,50	3,00	No	No	No	
63,90	5,10	10,80	Yes	Yes	No	No
65,40	4,70	3,80	No	No	No	
60,00	4,50	7,10	Yes	Yes	No	No
55,80	4,10	72,80	No	No	No	
56,90	4,30	5,80	No	No	No	
56,90	4,30	3,80	No	No	No	
62,70	4,20	7,30	No	No	No	
55,90	4,30	8,90	No	No	No	
60,60	5,60	7,60	Yes	No	Yes	No
59,70	4,30	4,60	No	No	No	
64,70	4,20	6,30	No	No	No	
57,80	5,00	3,60	Yes	No	Yes	No
55.4	4,60	8,60	No	No	No	
41,10	4,80	9,20	Yes	Yes	No	No
60,40	5,20	6,24	Yes	No	Yes	No
63,90	4,50	3,60	No	No	No	

65,90	4,40	4,70	No	No	No	
60,10	4,10	7,20	No	No	No	
55,40	4,70	6,60	Yes	Yes	No	No
55,80	4,90	4,00	Yes	Yes	No	No
66,00	4,60	6,20	Yes	Yes	No	No
63,10	4,30	2,40	No	No	No	
59,30	5,10	19,20	Yes	No	Yes	No
64,70	4,40	6,00	No	No	No	
65,90	4,80	2,70	Yes	No	Yes	No
56,00	4,40	3,70	No	No	No	
61,70	4,50	4,40	No	No	No	
58,50	5,32	5,50	Yes	No	Yes	No
63,70	4,50	10,30	No	No	No	
63,80	4,70	9,00	Yes	No	Yes	No
63,50	4,40	9,50	No	No	No	
60,10	4,30	3,80	No	No	No	
62,60	4,50	2,50	No	No	No	
59,00	4,60	4,20	Yes	Yes	No	No
50,70	5,00	14,80	Yes	No	Yes	No
60,70	5,00	11,90	Yes	Yes	No	No
63,00	4,60	3,10	No	No	No	
59,30	4,40	5,20	No	No	No	
56,00	4,80	6,50	Yes	No	Yes	No
58,90	4,40	4,30	No	No	No	
63,30	4,50	8,50	No	No	No	
60,10	4,90	5,20	Yes	Yes	No	No
67,10	5,20	7,30	Yes	No	Yes	No
64,50	4,70	10,40	No	No	No	
62,00	4,50	8,50	No	No	No	
59,20	4,50	58,10	No	No	No	
55,30	4,30	4,20	No	No	No	
48,20	4,90	6,30	Yes	No	Yes	No
57,50	4,60	2,70	No	No	No	
45,60	4,50	4,40	Yes	Yes	No	No
51,00	4,80	5,50	No	No	No	
53,40	4,70	3,20	No	No	No	
68,50	4,70	11,60	Yes	Yes	No	No
60,10	4,60	2,24	No	No	No	
62,10	4,80	3,30	Yes	Yes	No	No
58,60	4,40	3,37	No	No	No	
58,60	4,80	3,40	Yes	No	Yes	No
55,10	4,40	2,40	No	No	No	
61,80	4,40	7,60	No	No	No	
56,20	4,70	5,30	No	No	No	
62,80	4,80	3,30	Yes	Yes	No	No
65,00	4,70	1,40	No	No	No	
57,20	4,30	2,00	No	No	No	
58,80	4,90	1,00	Yes	Yes	No	No
63,80	4,20	1,20	No	No	No	
57,30	4,40	1,70	No	No	No	
66,20	4,30	3,90	No	No	No	
62,50	4,30	2,10	No	No	No	
56,70	4,50	2,70	No	No	No	
61,40	4,60	1,00	Yes	Yes	No	No

55,40	4,40	2,10	No		No	No	
59,60	4,30	3,30	No		No	No	
63,80	4,30	3,30	No		No	No	
63,20	4,70	2,10	Yes		Yes	No	No
54,90	4,30	3,20	No		No	No	
63,00	4,20	3,10	No		No	No	
56,40	4,20	3,00	No		No	No	
49,50	4,20	3,50	No		No	No	
55,90	4,80	1,40	Yes		Yes	No	No
55,00	4,50	3,70	No		No	No	
67,40	4,40	10,70	No		No	No	
65,90	4,40	2,79	No		No	No	
50,30	4,40	5,60	No		No	No	
55,50	4,60	2,00	Yes		Yes	No	No
61,00	4,30	1,50	No		No	No	
61,20	4,30	2,20	No		No	No	
69,20	4,20	1,40	No		No	No	
67,20	4,20	1,40	No		No	No	
60,80	4,30	4,80	No		No	No	
55,60	4,00	1,40	No		No	No	
63,30	4,30	4,10	No		No	No	
56,80	4,50	2,00	No		No	No	
63,10	4,40	1,50	No		No	No	
64,80	4,10	1,90	No		No	No	
62,50	4,40	1,50	No		No	No	
62,90	4,50	2,10	No		No	No	
62,20	4,20	2,00	No		No	No	
61,60	4,40	3,30	No		No	No	
61,70	4,90	1,90	Yes		Yes	No	No
60,70	4,40	1,30	No		No	No	
64,50	4,50	3,00	No		No	No	
61,40	4,50	2,80	No		No	No	
61,90	4,40	1,80	No		No	No	
56,30	4,30	1,80	No		No	No	
62,30	4,60	2,00	No		No	No	
67,50	4,30	2,20	No		No	No	
64,50	4,30	1,80	No		No	No	
55,70	4,00	1,90	No		No	No	
62,50	4,30	1,40	No		No	No	
55,10	4,30	1,25	No		No	No	
58,00	4,20	1,09	No		No	No	
56,70	4,40	2,20	No		No	No	
60,10	4,70	1,70	No		No	No	
68,90	4,50	6,80	No		No	No	
62,10	4,70	2,10	No		No	No	
60,70	4,50	1,50	No		No	No	
50,70	4,20	1,80	No		No	No	

59,30	4,30	1,00	No	No	No
59,10	4,10	1,20	No	No	No

Medication
